# Editorial: Exploring the multidisciplinary roles of sports events

**DOI:** 10.3389/fspor.2026.1954895

**Published:** 2026-10-01

**Authors:** Maria João Custódio, Fernando Perna, Azila Azmi, Luís Carvalhinho, Elsa Pereira

**Affiliations:** 1 ESGHT, Universidade do Algarve, Faro, Portugal; 2 Centro de Investigação Desenvolvimento e Inovação em Turismo (CiTUR), Faro, Portugal; 3 Universiti Teknologi MARA Cawangan Pulau Pinang, Pulau Pinang, Malaysia; 4 Escola Superior de Desporto de Rio Maior / Instituto Politécnico de Santarém, Rio Maior, Portugal; 5 Sport Physical Activity and Health Research & Innovation Center (SPRINT), Rio Maior, Portugal

**Keywords:** event legacy, multidisciplinary approach, quality of life, social impact, sport marketing, sports events, volunteer management

The increasing complexity and diversity of sport events require corresponding advances in the ways they are observed and analysed within the scientific literature. A key challenge is the transition from predominantly economic evaluation approaches toward multidimensional frameworks that integrate economic, social, tourism, environmental, and territorial dimensions. Such frameworks are essential for understanding and assessing the role of the multiple stakeholders involved across different spatial scales (from local to international) and temporal horizons (from short- to long-term). For example, the systematic review of the literature by Rossini et al. (1) highlights the variability of the impacts of events of different dimensions on participants, host communities and destinations, a reality that can only be understood through observation/evaluation models that integrate economic, social and tourism indicators. Similarly, Bourbillères and Djaballah (2) reinforce the convergence towards a holistic approach, where understanding the impacts and legacy of events requires an articulated analysis of the economic, social, environmental and territorial dimensions. This integrated and multidisciplinary perspective provides the foundation for the Research Topic Exploring *the Multidisciplinary Roles of Sports Events*. This edition seeks to advance robust and interdisciplinary knowledge that can support evidence-based strategic decision-making in the planning, management, evaluation, and long-term legacy of sport events.

An introductory dimension of this research topic is volunteering as an engine of social legacy, with primary quantitative research on the Special Olympics World Games 2023 (SOWG 23) demonstrating that participation is driven primarily by intrinsic motivations, such as expressing personal values and seeking interpersonal contact. A parental history of volunteering is identified as a crucial predictor of long-term engagement, suggesting that values instilled during childhood shape adult commitment and requiring organisations to adopt tailored digital communication strategies to foster lasting engagement across diverse demographic groups. The subjectivity of quality perception further illustrates the multidisciplinary nature of events, where Service Quality and Role theories explain how different stakeholders evaluate “success”. Analysis reveals an “expectation–performance gap": active participants often perceive lower service quality than organisers or group leaders due to greater performance pressure and more stringent logistical requirements. Consequently, sports management must move toward a tailored experience design that addresses the specific needs of athletes, families, and spectators separately to promote long-term loyalty. Similarly, sporting events serve as a cultural battlefield for national identity, where qualitative content analysis of media narratives on the naturalisation of football players reveals a tension between pragmatic nationalism and cultural essentialism. By distinguishing between legal “thin citizenship” and cultural “thick citizenship,” the research highlights how the media act as mediators of public perception, defining the symbolic boundaries of an “imagined community” in an era of global talent migration. From a marketing lens, sponsorship effectiveness is determined by the “fit” or congruity between the brand and the event. A meta-analytic review of 31 studies confirms that high levels of fit generate positive cognitive and emotional responses, though these are moderated by cultural contexts; Eastern cultures prioritise emotional harmony and collectivism, while Western contexts see a stronger impact on behavioural intentions. Finally, the impact and legacy on Quality of Life (QOL) is examined through a scoping review that identifies a significant shift from evaluating purely economic outcomes toward measuring subjective well-being. The findings suggest that while one-off mega-events offer temporary “feel-good” effects, recurring participatory events foster more sustainable community cohesion. To advance this field, researchers advocate integrating objective physiological measures, such as Heart Rate Variability (HRV), to complement subjective surveys. Ultimately, the integration of these diverse methodologies proves that sports event success is a multidimensional construct requiring a roadmap that balances marketing strategies, human capital management, and social wellbeing.

In conclusion, the articles in this Research Topic offer a profound and critical perspective on the role of sport in contemporary society. Collectively, they provide academics, event managers, and policymakers with practical insights and actionable tools to maximise the social legacies of sports events by understanding the intergenerational dynamics of volunteer motivation, improve service delivery through greater recognition of the role-specific expectations of diverse event participants, navigate the complex identity politics surrounding sports journalism and national team recruitment, optimise sponsorship strategies by acknowledging cultural differences in consumer behaviour, and promote long-term societal wellbeing by moving beyond short-term economic metrics towards more holistic quality-of-life indicators. Taken together, the findings presented in this volume constitute a significant contribution to the ongoing dialogue on how sports events can be strategically leveraged to foster more inclusive, prosperous, and happier societies.
